# Who Needs Most? Multicenter Subanalysis of Blood Transfusion Profiles in the German Patient Blood Management Network Registry

**DOI:** 10.3390/jcm15051759

**Published:** 2026-02-26

**Authors:** Florian Rumpf, Suma Choorapoikayil, Lotta Hof, Denana Mehic, Philipp Helmer, Benedikt Schmid, Kai Zacharowski, Patrick Meybohm, German Patient Blood Management Network Collaborators

**Affiliations:** 1 Department of Anaesthesiology, Intensive Care, Emergency and Pain Medicine, University Hospital Würzburg, 97080 Würzburg, Germany; 2 Department of Anaesthesiology, Intensive Care Medicine and Pain Therapy, University Hospital Frankfurt, Goethe University Frankfurt, 60596 Frankfurt, Germany

**Keywords:** blood transfusion, Patient Blood Management (PBM), clustering, hemostasis

## Abstract

**Background**: Blood transfusion practices have evolved significantly in order to enhance patient care. The optimal strategies for administering red blood cell (RBC) transfusions is becoming rather clear; however, a comprehensive understanding of patients requiring transfusions of other blood components remains inadequate, leading to variability in clinical practice and outcomes. Here we examine surgical patients that could benefit from perioperative risk stratification. **Study Design and Methods**: We analyzed subgroups of a prospective, multicenter follow-up study and identified three distinct transfusion profiles across surgical disciplines: low (n = 1,035,588, 92.0%), moderate (n = 81,243, 7.2%), and high (n = 8413, 0.7%). These profiles are characterized by varying requirements for RBC, plasma, and platelet units. **Results**: While most patients were clustered in the low transfusion profile, blood component use only increased significantly in the high transfusion profile. Notably, patients in the high transfusion profile benefited from Patient Blood Management (PBM) interventions with a reduction of the predefined composite endpoint of in-hospital mortality and postoperative complications (ischemic stroke, myocardial infarction, pneumonia, sepsis and acute renal failure with renal replacement therapy) from 28.2% to 26.0% and an OR of 0.90 (95% CI 0.80–1.00, *p* = 0.048) compared to the moderate transfusion profile. Conversely, the low transfusion profile encompassed patients with minimal transfusion needs, presenting opportunities to refine resource allocation and risk stratification. **Discussion**: These findings underscore the potential for improving patient outcomes and indicate that implementing targeted PBM interventions can reduce the risk of adverse events and mortality. This study advances the field by identifying specific transfusion profiles that can guide future research and clinical practices towards more personalized and efficient blood management in perioperative care.

## 1. Introduction

The advent of component therapy, where blood is separated into its components such as red blood cells (RBCs), plasma, platelets and coagulation factors, has revolutionized hemotherapy, moving beyond the traditional use of whole blood to a more targeted and efficient transfusion strategy [1]. Blood transfusions are now pivotal in modern medicine, serving critical roles in trauma care, surgical interventions, and managing chronic diseases like anemia, cancer, and hematological disorders [2]. With the increasing complexity of patient care, particularly in surgery, the demand for transfusion strategies that meet individual patient needs is more crucial than ever. With RBC transfusions, there is a clear objective to improve oxygen delivery to the tissues. Plasma, platelets and individual coagulation factors can be indicated in the bleeding patient with the objective of hemostasis but it becomes more difficult to judge the right therapeutic course of action. The timely and appropriate administration of blood products is crucial and potentially life-saving for the bleeding patient [3].

Blood transfusion recipient characteristics have been described previously [4,5] but a holistic understanding of patients with high blood transfusion requirements in the operative setting is lacking. While some variability in transfusion requirements can be explained by the extent of a surgical procedure and center-specific factors, the variation in non-RBC transfusion requirements between surgical specialties needs to be better understood. Furthermore, many healthcare providers face uncertainties concerning the optimal combination and timing of blood product use, leading to variability in clinical practice and possible adverse patient outcomes [6,7]. This underscores the need for investigations to support evidence-based decision-making in blood component transfusion, particularly in emergencies where both oxygen delivery and prompt hemostatic resuscitation are critical.

The practical implementation of blood product administration demands meticulous planning and effective resource management to ensure timely access to necessary components. Strategic resource allocation is crucial in perioperative medicine to balance the immediate availability of blood products while avoiding waste and inefficiency [8]. High-risk patients—such as those with coagulopathies, significant comorbidities, or undergoing extensive procedures—have been suggested to have the greatest benefit from targeted interventions [9].

These aspects are unified in Patient Blood Management (PBM) as an essential framework in contemporary perioperative medicine, focusing on improving patient outcomes by optimizing hemostasis, conserving blood, and refining transfusion practices [10]. This comprehensive approach emphasizes the balance between preserving sufficient blood volume and reducing unnecessary blood loss during surgery. Implementing effective PBM strategies not only enhances patient safety but also supports better recovery by reducing the risks of anemia, transfusion-related complications, and extended hospital stays [11].

In this study, we aimed to identify different profiles of blood transfusion recipients. We assessed the distribution of blood products among surgical patients to identify subgroups with high transfusion requirements. Therefore, patients that would benefit from perioperative risk optimization and specifically PBM strategies could be identified.

## 2. Methods

### 2.1. Study Population

This study included patients aged ≥18 years who underwent surgery and were discharged from 14 German hospitals between 1 January 2010, and 31 December 2019 as part of the German Patient Blood Management Network follow-up study. Detailed methods on this prospective, multicenter, observational, controlled cohort study with a before-and-after design have been published previously [12]. Briefly, anonymized routine data were gathered from the electronic systems of participating hospitals, and supplemented with pharmacy and blood bank data by the local information technology officers. Routine error checks and validation were performed by center-specific experts and PBM Network biostatisticians.

Blood components were produced and transfused in accordance with the hemotherapy guideline of the Federal Medical Association (“Bundesärztekammer”) [13]. For the purpose of this study patients that did not have PCC and fibrinogen units reported were excluded from the analysis (n = 51,612). Furthermore, the smallest surgical category with significant overlap of primary ICU patients was removed (n = 24,961).

### 2.2. Outcome Measures

The analyzed outcomes were the same predefined outcome measures included in the prospectively designed, multicenter follow-up study [12]. Specifically, this study examined transfusion rates, mean blood product consumption per 1000 patients and a binary composite outcome of in-hospital mortality and postoperative complications (ischemic stroke, myocardial infarction, pneumonia, sepsis and acute renal failure with renal replacement therapy).

### 2.3. Ethics

Approval for the study was obtained by the leading Ethics Committee of the University Hospital Frankfurt (Reference 318/17), by the ethics committees of all participating centers, and by the Hessian data protection officer (Reference 43.60; 60.01.21-ga; 24 October 2018). Any written informed consent of patients was waived by the Ethics Committee.

### 2.4. PBM Intervention

Details for the PBM intervention in the German Patient Blood Management Network follow-up study have been published before [12]. In summary, all patients received standard local routine care as determined by the attending physicians. Additionally, a PBM program was implemented that emphasized three key pillars: preoperative optimization of hemoglobin levels, blood-saving techniques, and adherence to transfusion guidelines and standardization of allogeneic blood component transfusion.

### 2.5. Data Sharing Statement

For original data, please contact patientbloodmanagement@unimedizin-ffm.de.

### 2.6. Statistics

The consumption of blood components was measured in units where appropriate. For primary blood components (RBCs, platelets and plasma), hemotherapy was recorded as the number of transfused units, such as one bag of packed RBC. Fibrinogen usage was measured in grams, while Prothrombin complex concentrate (PCC) usage was quantified in thousands of international units (IU). Ratios of blood products were calculated on a per-patient basis and then given as means with standard deviation for subgroups. Surgeries were categorized by surgical discipline according to procedure records. Detailed information about the procedures was recorded using electronic operation and procedure codes (OPS). In Germany, OPS codes are highly reliable for documenting medical procedures as they are managed at the national level for purposes like billing, documentation, and statistical analysis. For comorbidities and postoperative complications encoded diagnosis were used as defined by the International Classification of Diseases (ICD).

To identify distinct transfusion profiles for surgical patients, we used unsupervised machine learning algorithms to perform two-step clustering. First, sparse kmeans clustering was performed on encoded procedures and diagnosis to identify procedural similarities. This yielded 50 clusters that were then included in step two of the clustering method. Ward’s hierarchical agglomerative clustering method was used with a distance measure optimized for counts [14]. It aims at finding compact, spherical clusters in the sample based on the procedural clusters and the number of transfused RBC, plasma and platelet units.

For statistical modeling, confounders were selected by acyclic graphing based on literature and expert consensus. For the composite outcome estimates were derived with generalized linear mixed-effects models with age, sex, comorbidities and clusters as fixed effects and both center and type of surgery as random effects. Random-effect variables captured baseline risk differences attributable to between-center variability in case volume, specialization, patient mix, and local clinical practice patterns. The PBM intervention was modeled conditionally within clusters to allow the effect to differ by cluster. Additionally, center-specific effects of the PBM intervention were modeled to account for heterogeneity in how PBM associates with outcomes across centers. This approach accounts for any interaction between the PBM intervention and cluster- or center-specific effects. Acyclic graphs and model diagnostics did not identify any additional relevant interactions to account for. Model estimates were then derived as odds ratios (OR) and given with profile likelihood-based 95% confidence intervals (95% CI). Based on the model estimates, we also calculated outcome probabilities for the non-reference groups. Statistical analysis and graphing were performed using R software version 4.3.4. The study was documented in accordance with the Reporting of Observational Studies in Epidemiology (STROBE) guidelines [15].

## 3. Results

Of 1,125,244 included surgical patients, 119,369 (10.6%) received at least one blood product during hospital stay. From 2010 to 2019, the predominant blood product administered was RBC units, totaling 564,220 units, with plasma at 229,817 units and platelets at 120,365 units following behind.

**Figure 1:**
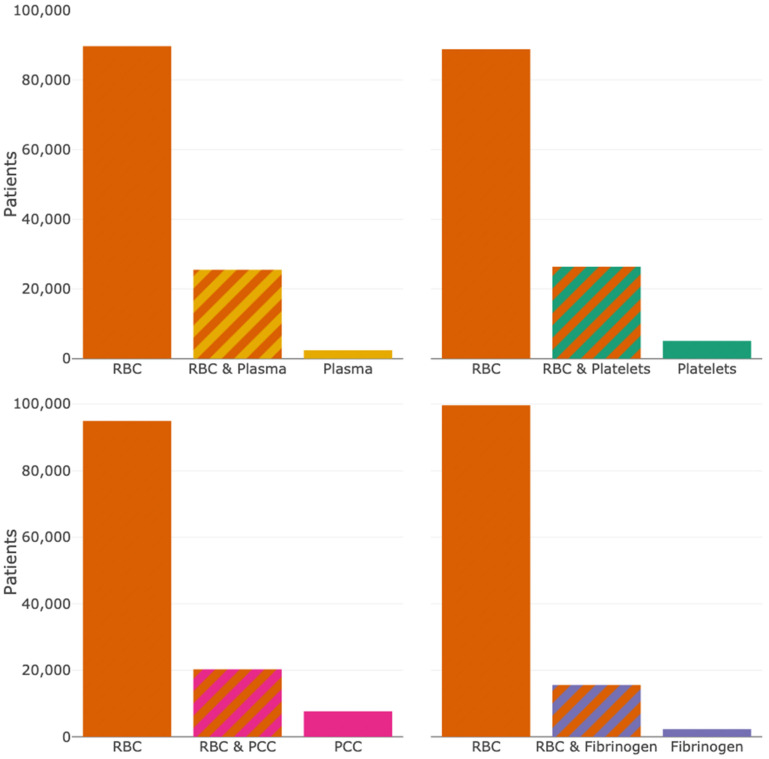
Variations in blood product combinations. Consumption of non-red blood cell (RBC) units alone or in conjunction with RBC units. More details are illustrated interactively at wue-ains.shinyapps.io/blood-products/, accessed on 24 February 2026.

In total, 105,583 patients (9.4%) received RBC transfusion. Of these patients 66,196 (5.9%) received RBC units only, 21,211 (1.9%) received RBC units in combination with plasma, 22,009 (2.0%) received RBC units in combination with platelets, 12,235 (1.1%) received RBC units in combination with fibrinogen and 16,686 (1.5%) received RBC units in combination with PCC (Figure 1). Less commonly, plasma (2368, 0.2%), platelets (5050, 0.4%) fibrinogen (2241, 0.2%), and PCC (7452, 0.7%) were transfused without RBC units.

Hemostatic resuscitation was overall more plasma-based than coagulation factor-based. Fibrinogen without plasma was transfused in 7391 (0.7%), while plasma without fibrinogen was transfused in 16,494 (1.5%). Patients that received plasma often additionally received fibrinogen 7085 (0.6%). PCC without plasma was administered in 16,316 patients (1.4%), and almost equally as frequently, plasma was given without PCC in 15,757 patients (1.4%). The combination of PCC and plasma was administered in 7822 (0.7%).

### 3.1. Distribution of Transfused Blood Components According to Type of Surgery

**Figure 2:**
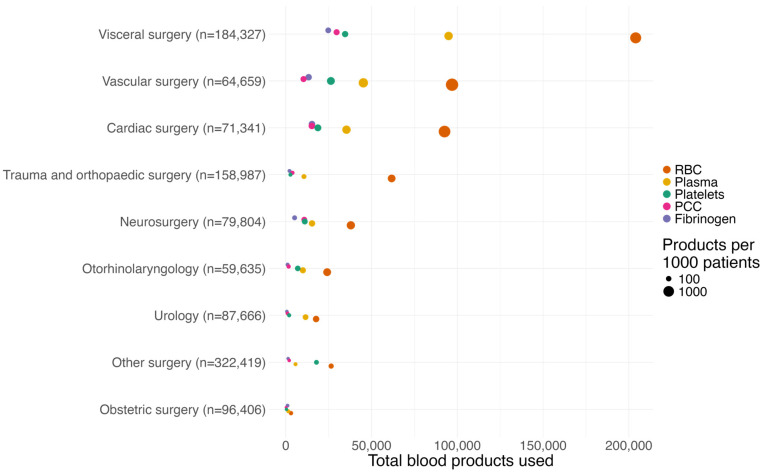
Distribution of transfused blood products according to type of surgery. In visceral surgery, 203,983 RBC units, 94,891 plasma units, 34,566 platelet units, 29,609,043 IE of PCC and 24,733 g of fibrinogen were transfused. Per 1000 patients, the mean transfusion rate is 1499 (±39) for RBCs, 699 (±51) for plasma, 406 (±21) for platelets, 160 (±7) for PCC and 206 (±10) for fibrinogen. See Supplementary Materials Tables S2 and S3 for underlying data.

The highest number of RBC and plasma units were used for patients undergoing visceral surgery, followed by vascular and cardiac surgery (Figure 2, Supplementary Materials Tables S1–S3). The number of transfused RBC units per 1000 patients was highest in patients undergoing vascular surgery (1499 ± 39) and cardiac surgery (1297 ± 24). Preoperative anemia increased the risk of RBC transfusion across all surgical disciplines (Supplementary Material Figure S2).

### 3.2. Distribution of Transfused Blood Components According to Age and Sex

Overall, the number of transfused blood products continuously increased until the age of 75 years. In trauma and orthopedic surgery, more RBC units were transfused in female patients compared to male patients of similar age, whereas in visceral and cardiac surgery more RBC units were transfused in male than female patients. This pattern was also observed for the number of transfused plasma, platelets, PCC and fibrinogen units and was especially distinct in cardiac surgery. For RBC, plasma and fibrinogen a distinct proportion of units was transfused in women of childbearing age during obstetric surgery (Supplementary Material Figure S1).

### 3.3. Transfusion Profiles

**Figure 3:**
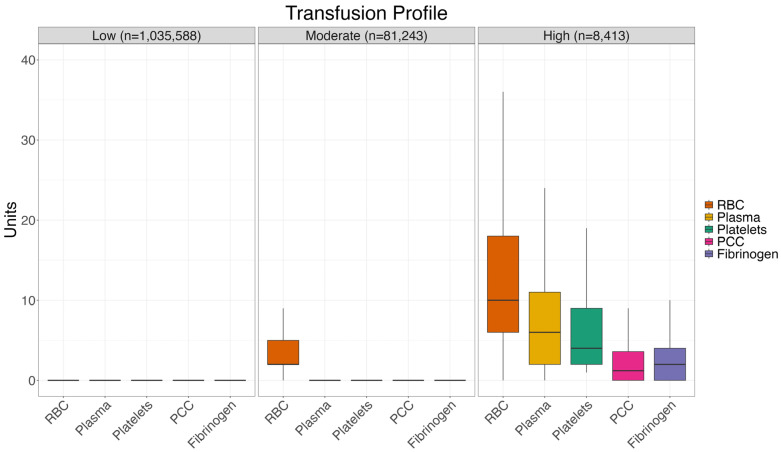
Distribution of transfused blood products according to transfusion profile.

Within our cohort, we identified three subgroups of patients with distinct low, moderate, and high transfusion profiles (Table 1). The moderate transfusion profile included 81,243 patients (7.2%) who required primarily RBC transfusions with a median of 2 RBC units (Figure 3). While these individuals required transfusions more frequently than those in the low transfusion profile, they did not demonstrate the exceptionally high levels of transfusion demand observed in the high transfusion profile. The moderate transfusion profile group were used as the reference group and compared with low and high transfusion profile group.

In the high transfusion profile (n = 8413, 0.7%), patients exhibited elevated transfusion requirements across various blood products. These individuals presented with the most complex transfusion needs, typically associated with scenarios requiring mass transfusion protocols. In this group, targeted hemostatic therapy had great therapeutic importance: 3971 (47.2%) patients received both plasma and fibrinogen while fibrinogen without plasma was used in only 355 (4.2%) patients. Similarly, 4208 (50.0%) of patients received both plasma and PCC while only 467 (5.6%) patients received PCC without plasma. The high transfusion profile patients mostly underwent visceral, vascular or cardiac surgery. The median plasma and platelet ratios for high transfusion profiles in the cohort were 0.6 (0.1; 1.1) and 0.4 (0.2; 0.9). Compared to the moderate transfusion profile, the composite endpoint was observed with an OR of 3.35 (95% CI 3.08–3.63, *p* < 0.001). In this high-risk setting, the PBM intervention was able to reduce the composite endpoint with an OR of 0.90 (95% CI 0.80–1.00, *p* = 0.048). The probability of the composite endpoint could be reduced from 28.2% to 26.0%.

The low transfusion profile (n = 1,035,588, 92.0%) primarily consisted of individuals with very infrequent needs for RBC transfusions and even more rare requirements for other blood products. Their overall reliance on transfusions was very low compared to the other subgroups. Compared to the moderate transfusion profile, the composite endpoint was much less frequent with an OR of 0.12 (95% CI 0.11–0.12, *p* < 0.001). For these patients, the PBM intervention did not affect the composite endpoint with OR 1.02 (95% CI 0.97–1.08, *p* = 0.490) and a very low endpoint probability of 1.4% in both the PBM and non-PBM groups.

## 4. Discussion

This study describes different blood transfusion profiles in surgical patients. We identified a high blood transfusion profile with increased risk for multiple RBC transfusions in combination with other blood components. These patients often received plasma in combination with individual coagulation factors for hemostatic resuscitation. Noticeably, they benefited most from a PBM intervention. The low transfusion profile had a distinctly low risk of both blood component need and perioperative morbidity and mortality. The profile embodies the elective surgical patient with no comorbidities. The identification of blood transfusion profiles could offer potential for risk stratification and improved resource allocation. While the low transfusion profile made up the majority of surgical patients, this group hardly needed any blood components. Meanwhile, the moderate and high transfusion profiles received a majority of the RBC transfusions. Transfusions other than RBCs were almost exclusively observed in the high transfusion profile.

Studies for specific surgical procedures have shown a great range in blood transfusion needs [16]. For example, cardiac surgery often require significant amounts of blood transfusions owing to blood loss and coagulopathy [17]. Conversely, certain procedures, such as elective thyroid surgeries, show a markedly low requirement for blood transfusions [18]. This aligns very well with the blood transfusion profiles we have identified in this study. However, while there were differences in the type of surgery between transfusion profiles, a significant proportion (>5%) of patients in the low transfusion profile underwent cardiac surgery. There is also variability in transfusion practices across different centers, which underscores the importance of standardized preoperative and intraoperative practices to minimize unnecessary blood component use [19]. Inappropriate blood component use has been decreased in recent years mainly due to PBM and other initiatives [12].

For massive bleeding events, different strategies for hemostatic resuscitation have been investigated. Early military studies proposed that a high transfusion ratio of plasma and packed RBC, that corresponds more closely to whole blood, is associated with improved survival in patients who require massive transfusion [20]. Balanced transfusion strategies have since been applied and studied throughout military and civilian trauma systems [21,22]. The practice has been refuted due to the retrospective nature of the studies. They were majorly limited by survival bias, as patients with less severe injuries were more likely to obtain or reach balanced transfusion. This study does not aim to add to this debate and, thus, we refrained from reporting survival rates based on blood component ratios. In the trauma setting, prospective trials have not demonstrated improved survival with higher plasma and platelet ratios [23,24]. Only in the high transfusion profile did we identify a median plasma ratio that approaches 1:1. The platelet ratios were mostly below 1:1 across surgical disciplines, even for patients in the high transfusion profile.

Also stemming from military trauma resuscitation, whole blood transfusion has been increasingly evaluated in civilian trauma care and surgical settings. Compared with component therapy, whole blood can simplify logistics, shorten time to first transfusion, and provide red cells, plasma, and platelets in a single product, potentially mitigating dilutional coagulopathy and reducing donor exposure [25,26]. While this may be beneficial in selected settings, none of the centers in our study used it routinely; therefore, we were unable to evaluate the role of whole blood transfusion.

Hemostatic resuscitation strategies based on coagulation factors have been compared to plasma-based strategies in randomized controlled trials. First-line coagulation factor concentrates seem to be superior for the reversal of trauma-induced coagulopathy to plasma [27]. Studies on the effectiveness of balanced transfusion in elective surgery are quite limited, likely due to the lack of standardized transfusion practices across surgical subspecialties. A very recent trial has demonstrated that PCC has superior hemostatic efficacy and improved safety over plasma in bleeding cardiac surgery patients [28]. In our study, we found a mix of plasma- and coagulation factor-based strategies without any clear tendencies for either.

While transfusion strategies may improve survival, mass transfusion events remain highly dynamic and unpredictable, often requiring rapid decision-making and individualized treatment approaches [29]. This highlights the importance of maintaining a readily available inventory of blood components and having well-trained personnel to adapt to the evolving needs of the patient. Additionally, variability in the timing, ratio, and volume of blood components transfused during surgery underscores the need for continued research to optimize protocols [30]. This is particularly relevant in elective surgical settings, where proactive planning and patient-specific factors, such as comorbidities and coagulation status, can significantly influence transfusion practices and outcomes. We demonstrated in our study that we can identify patients with a high transfusion profile. This may be a foundation to improve risk stratification and resource allocation in this setting. Furthermore, we were able to demonstrate that a PBM intervention significantly improved morbidity and mortality in this subgroup. Importantly, this finding represents a cluster-conditional estimate and should be interpreted accordingly, rather than as definitive evidence of benefit across all patient groups.

There are limitations to this study: Subgroups of a prospective, multicenter follow-up study were retrospectively analyzed and, thus, selection bias and unmeasured confounders remain a possibility. However, all estimates presented in this study were adjusted for the measured confounders. There is also no assessment of post discharge morbidity and mortality, and future studies should evaluate these outcomes. Strengths of the study include its multicenter design, the uniquely large sample size and identification of transfusion profiles.

In conclusion, this study identified distinct blood transfusion profiles in surgical patients, highlighting a high transfusion profile with increased use of RBCs and other blood components. While the low transfusion profile represented the majority of patients with minimal blood product needs and low morbidity, the high transfusion profile accounted for most plasma and coagulation factor use and benefited significantly from PBM interventions. These findings suggest potential for risk stratification and improved resource allocation in perioperative care.

## Figures and Tables

**Table 1:** Cohort characteristics for derived clusters: Categorical variables (Sex, type of surgery) are presented as counts with the fractional percentage in parentheses. Continuous variables are given as the median with interquartile range in parentheses.

Variable	Low Transfusion Profile n = 1,035,588	Moderate Transfusion Profile n = 81,243	High Transfusion Profile n = 8413
Female	529,156 (51.1%)	35,039 (43.1%)	3070 (36.5%)
Age (years)	60 (41; 74)	69 (58; 77)	63 (53; 73)
Comorbidities	0 (0; 1)	1 (0; 2)	1 (0; 1)
Hb at admission (g/dL)	13.4 (12.1; 14.6)	11.5 (9.5; 13.3)	11.2 (9.2; 13.2)
Hb at discharge (g/dL)	12.2 (10.6; 13.7)	9.6 (8.6; 10.6)	9.3 (8.4; 10.4)
Red blood cells (units)	0.0 (0.0; 0.0)	2.0 (2.0; 5.0)	10.0 (6.0; 18.0)
Plasma (units)	0.0 (0.0; 0.0)	0.0 (0.0; 0.0)	6.0 (2.0; 11.0)
Platelets (units)	0.0 (0.0; 0.0)	0.0 (0.0; 0.0)	4.0 (2.0; 9.0)
Fibrinogen (g)	0.0 (0.0; 0.0)	0.0 (0.0; 0.0)	2.0 (0.0; 4.0)
PCC (IU)	0.0 (0.0; 0.0)	0.0 (0.0; 0.0)	1200.0 (0.0; 3600.0)
Plasma ratio	0.0 (0.0; 0.0)	0.0 (0.0; 0.0)	0.6 (0.1; 1.1)
Platelet ratio	0.0 (0.0; 0.0)	0.0 (0.0; 0.0)	0.4 (0.2; 0.9)
Visceral surgery	156,121 (15.1%)	25,534 (31.4%)	2672 (31.8%)
Vascular surgery	49,080 (4.7%)	13,393 (16.5%)	2186 (26.0%)
Cardiac surgery	54,684 (5.3%)	14,751 (18.2%)	1906 (22.7%)
Trauma and orthopedic surgery	152,953 (14.8%)	5841 (7.2%)	193 (2.3%)
Neurosurgery	69,275 (6.7%)	10,211 (12.6%)	318 (3.8%)
Otorhinolaryngology	56,074 (5.4%)	3277 (4.0%)	284 (3.4%)
Urology	84,253 (8.1%)	3292 (4.1%)	121 (1.4%)
Other surgery	317,108 (30.6%)	4609 (5.7%)	702 (8.3%)
Obstetric surgery	96,040 (9.3%)	335 (0.4%)	31 (0.4%)

## Data Availability

The data presented in this study are available on request from the corresponding author.

## Supplementary Materials

The following supporting information can be downloaded at: https://www.mdpi.com/article/10.3390/jcm15051759/s1. Supplementary Table S1: Total number of patients transfused with blood products according to type of surgery; Supplementary Table S2: Total units of blood products transfused according to type of surgery; Supplementary Table S3: Units of transfused blood products per 1,000 patients according to type of surgery; Supplementary Figure S1: Distribution of transfused blood products according to age and sex; Supplementary Figure S2: Transfusion risk in patients with and without preoperative anemia by type of surgery.

## Appendix A

**Table A1:** German Patient Blood Management Network Collaborators.

Center	Collaborators
Klinikum Westmuensterland Bocholt	Olaf Baumhove, Samuel de Leeuw van Weenen (Department of Anaesthesiology, Intensive Care Medicine and Pain Therapy).
University Hospital Bonn	Markus Velten, Maria Wittmann, Claudia Neumann, Andrea Kirfel, Nadine Straßberger-Nerschbach, Heidi Ehrentraut, Daniel Grigutsch, Vera Guttenthaler, Alma Puskarevic, Ghaith Mohssen (Department of Anaesthesiology and Operative Intensive Care Medicine), Johannes Oldenburg (Institute of Experimental Haematology and Transfusion Medicine), Jan Görtzen (Department of Internal Medicine I).
DONAUISAR Klinikum (Deggendorf & Dingolfing)	Diana Narita, Lighvani Barbara (Institute of Laboratory Diagnostics und Transfusion Medicine), Josef Michael Huber (Institute of Laboratory Diagnostics, Immunohematology and Microbiology).
University Hospital Frankfurt	Lea Valeska Blum, Suma Choorapoikayil, Lotta Hof, Vanessa Neef, Kai Zacharowski (Department of Anaesthesiology, Intensive Care Medicine and Pain Therapy), Thomas Walther, Harald Keller (Department of Thoracic and Cardiovascular Surgery), Andreas Schnitzbauer (Department of General and Visceral Surgery), Thomas Schmitz-Rixen, Kyriakos Oikonomou (Department of Vascular and Endovascular Surgery), Bjoern Steffen (Department of Haematooncology), Stefan Zeuzem (Department of Gastroenterology and Hepatology), Marcus Czabanka (Department of Neurosurgery), Felix Chun (Department of Urology), Ingo Marzi, (Department of Trauma, Hand and Reconstructive Surgery), Timo Stöver (Department of Ear, Nose and Throat), Shahram Ghanaati (Department of Oral Maxillofacial and Plastic Facial Surgery), Frank Louwen, (Department of Gynecology and Obstetrics).
Goethe University Frankfurt	Markus M. Mueller, Christoph Geisen, Erhard Seyfried (German Red Cross Blood Transfusion Service Baden-Wuerttemberg–Hessen, Institute of Transfusion Medicine and Immunohematology), Eva Herrmann (Institute of Biostatistics and Mathematical Modelling, Department of Medicine).
Agatharied Hospital Hausham	Alexandra Bayer (Department of Anaesthesiology and Intensive Care Medicine).
SLK-Kliniken Heilbronn	Henry Weigt, Björn Lange (Department of Anaesthesiology).
University Hospital Jena	Ansgar Raadts (Department of Anaesthesiology and Intensive Care Medicine), Christoph Haas (Executive Department for Structure, Process and Quality Management).
University Medical Center Schleswig-Holstein	Matthias Gruenewald, Gunnar Elke, Johannes Duemmler, Ulf Lorenzen (Department of Anaesthesiology and Intensive Care), Matthias Pagel (Information-Technology UKSH), Thomas Puehler (Department of Cardiovascular Surgery), Julius Pochhammer (Department of General and Visceral Surgery), Tim Klueter (Department of Trauma, Hand and Reconstructive Surgery), Hajrullah Ahmeti (Department of Neurosurgery), Dirk Bauerschlag (Department of Gynaecology and Obstetrics), Henning Wieker (Department of Oral Maxillofacial and Plastic Facial Surgery), René Rusch (Department of Vascular Surgery).
Klinikum Leverkusen	Jens Friedrich, Gerd Molter (Department of Anaesthesiology and Intensive Care Medicine).
München Klinik (Bogenhausen)	Brigitte Bräutigam (Business Unit Hospital Controlling) Patrick Friederich (Department of Anaesthesiology, Critical Care Medicine, Pain Therapy).
University Hospital Muenster	Dana Janina Jenke, Kira Kieserling, Dennie Scholle, Andrea U. Steinbicker, Alexander Zarbock (Department of Anaesthesiology, Intensive Care Medicine and Pain Therapy), Sven Martens (Department of Thoracic and Cardiovascular Surgery), Andres Schrader (Clinic for Urology and Pediatric Urology), R. G. Geissler, H. Hillmann (Institute of Transfusion Medicine), Georg Lenz (Medical Clinic A, Haematology, Pneumology, Haemostaseology and Oncology).
Diakonie Hospital Nuremberg	Klaus Schwendner (Department of Anaesthesiology and Operative Intensive Care Medicine).
Ortenau Klinikum Offenburg-Gengenbach/Kehl	Lukas Spitzmüller, Josef Thoma, Viola Weber (Department of Anesthesiology and Operative Intensive Care Medicine).
University Hospital Würzburg	Florian Rumpf, Philipp Helmer, Sebastian Hottenrott, Peter Kranke, Patrick Meybohm, Daniel Roeder, Magdalena Sitter, Benedikt Schmid, Tobias Haas, Michael Kotlyar (Department of Anaesthesiology, Intensive Care, Emergency and Pain Medicine).
