# Supplementary material for: Who Needs Most? Multicenter Subanalysis of Blood Transfusion Profiles in the German Patient Blood Management Network Registry
Source: J Clin Med. 2026 Feb 26;15(5):1759. doi: 10.3390/jcm15051759 (PMC12985576; doi:10.3390/jcm15051759)

Journal of
Clinical Medicine

MDPI

Article

Who Needs Most? Multicenter subanalysis of blood transfusion profiles in the German Patient Blood Management Network Registry

Florian Rumpf [1,✉], Suma Choorapoikayil [2], Lotta Hof [2], Denana Mehic [2], Philipp Helmer [1], Benedikt Schmid [1], Kai Zacharowski *[,2], Patrick Meybohm *[,1] and German Patient Blood Management Network Collaborators [3]

Supplementary tables

Supplementary table 1: Total number of patients transfused with blood products according to type of surgery.

Surgical discipline	RBC	Plasma	Platelets	PCC	Fibrinogen
Visceral surgery	28,043 (15.2%)	7,723 (4.2%)	5,662 (3.1%)	6,615 (3.59%)	3,599 (1.95%)
Cardiac surgery	21,551 (30.2%)	5,857 (8.2%)	8,250 (11.6%)	7,039 (9.87%)	4,743 (6.65%)
Trauma and orthopaedic surgery	16,263 (10.2%)	1,642 (1.0%)	802 (0.5%)	1,516 (0.95%)	625 (0.39%)
Vascular surgery	15,667 (24.2%)	4,095 (6.3%)	5,466 (8.5%)	3,638 (5.63%)	3,215 (4.97%)
Neurosurgery	9,126 (11.4%)	2,273 (2.8%)	3,077 (3.9%)	3,675 (4.61%)	1,292 (1.62%)
Other surgery	5,583 (1.7%)	551 (0.2%)	2,495 (0.8%)	680 (0.21%)	303 (0.09%)
Urology	4,794 (5.5%)	651 (0.7%)	406 (0.5%)	423 (0.48%)	187 (0.21%)
Otorhinolaryngology	3,265 (5.5%)	569 (1.0%)	741 (1.2%)	462 (0.77%)	182 (0.31%)
Obstetric surgery	1,291 (1.3%)	218 (0.2%)	160 (0.2%)	90 (0.09%)	330 (0.34%)
Total	105,583 (9.4%)	23,579 (2.1%)	27,059 (2.4%)	24,138 (2.1%)	14,476 (1.3%)

Supplementary table 2. : Total units of blood products transfused according to type of surgery.

Surgical discipline	RBC	Plasma	Platelets	PCC	Fibrinogen
Visceral surgery	203,983	94,891	34,566	29,609	24,733
Vascular surgery	96,915	45,199	26,273	10,319	13,290
Cardiac surgery	92,537	35,358	18,686	15,171	15,238
Trauma and orthopaedic surgery	61,698	10,581	2,688	3,861	2,134
Neurosurgery	37,908	15,283	11,045	10,753	5,112
Other surgery	26,425	5,647	17,854	1,967	1,323
Otorhinolaryngology	24,095	9,934	6,946	1,638	1,028
Urology	17,646	11,487	1,929	973	600
Obstetric surgery	3,013	1,437	378	196	1,000

Surgical discipline	RBC	Plasma	Platelets	PCC	Fibrinogen
Total	564,220	229,817	120,365	74,487	64,458

Supplementary table 3. : Units of transfused blood products per 1,000 patients according to type of surgery.

Surgical discipline	RBC	Plasma	Platelets	PCC	Fibrinogen
Vascular surgery	1,499 (±39)	699 (±51)	406 (±21)	160 (±7)	206 (±10)
Cardiac surgery	1,297 (±24)	496 (±19)	262 (±9)	213 (±6)	214 (±7)
Visceral surgery	1,107 (±24)	515 (±23)	188 (±10)	161 (±7)	134 (±7)
Neurosurgery	475 (±19)	192 (±13)	138 (±9)	135 (±6)	64 (±8)
Otorhinolaryngology	404 (±22)	167 (±35)	116 (±17)	27 (±4)	17 (±5)
Trauma and orthopaedic surgery	388 (±9)	67 (±5)	17 (±2)	24 (±2)	13 (±1)
Urology	201 (±8)	131 (±27)	22 (±4)	11 (±1)	7 (±1)
Other surgery	82 (±4)	18 (±4)	55 (±4)	6 (±1)	4 (±1)
Obstetric surgery	31 (±3)	15 (±9)	4 (±1)	2 (±1)	10 (±1)

Supplementary figures

Supplementary figure 1. : Distribution of transfused blood products according to age and sex.

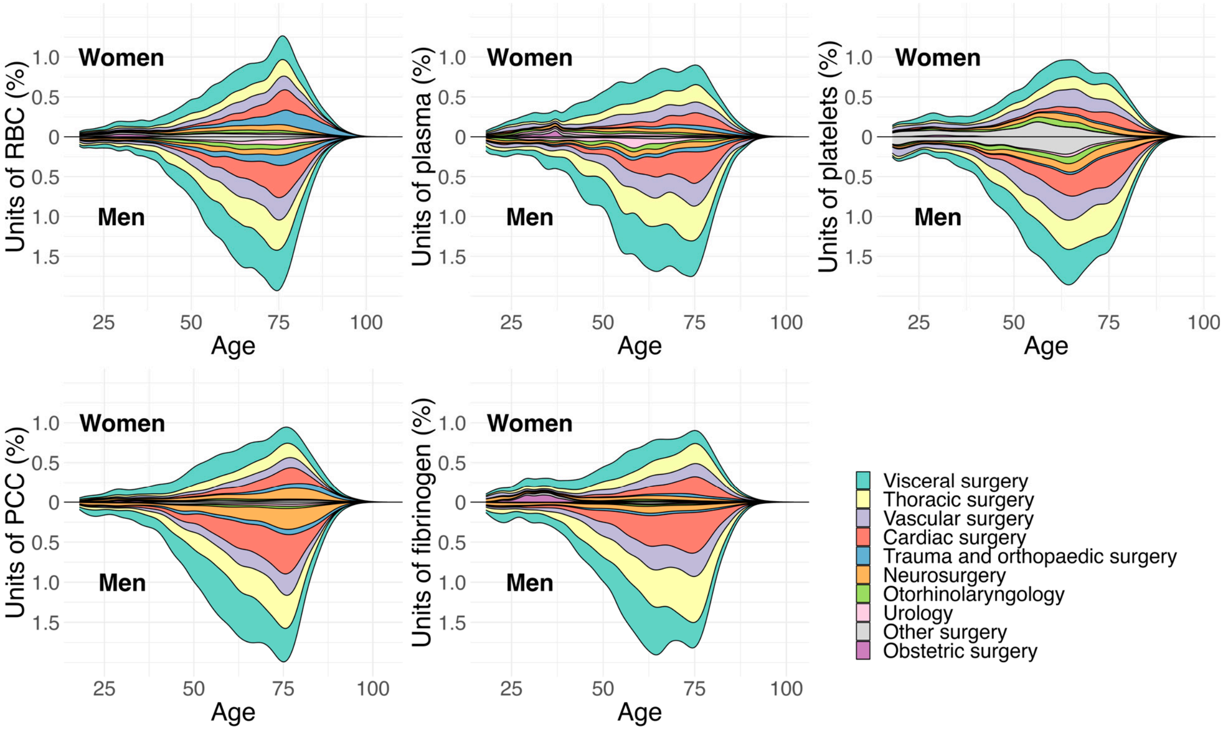

Supplementary figure 2. : Transfusion risk in patients with and without preoperative anemia by type of surgery.

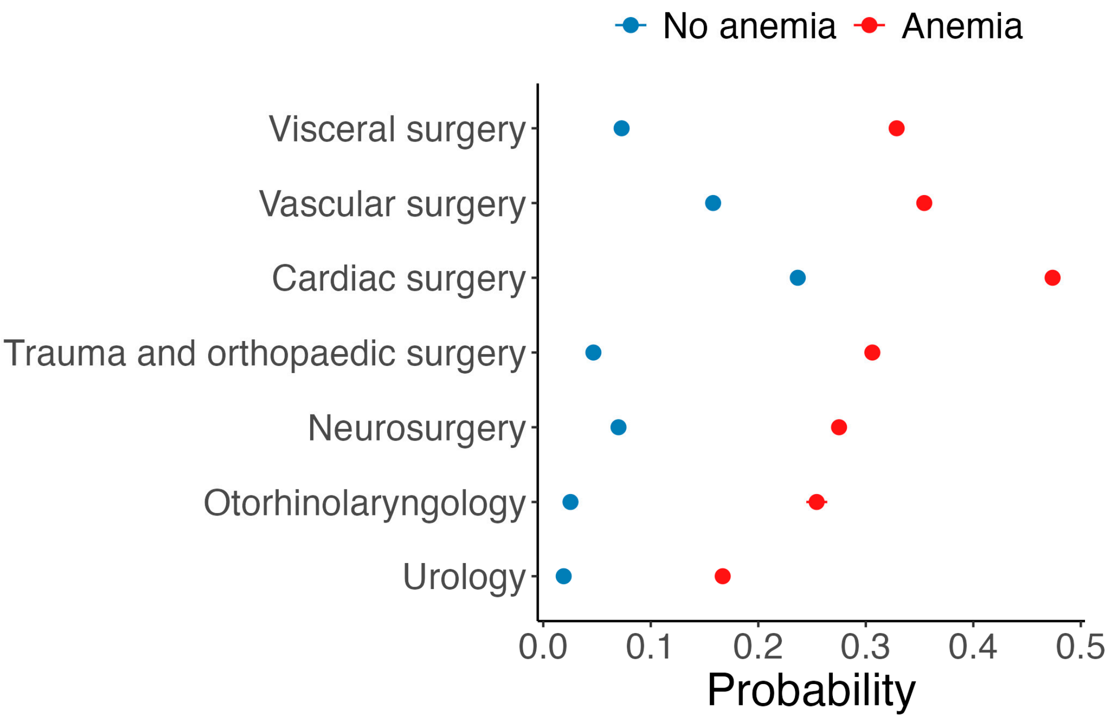

Supplement: Supplementary file 1 [file jcm-15-01759-s001.zip › jcm-4085159-supplementary.pdf]
